# Validation of Transient Elastography and Comparison with Spleen Length Measurement for Staging of Fibrosis and Clinical Prognosis in Primary Sclerosing Cholangitis

**DOI:** 10.1371/journal.pone.0164224

**Published:** 2016-10-10

**Authors:** Hanno Ehlken, Raluca Wroblewski, Christophe Corpechot, Lionel Arrivé, Tim Rieger, Johannes Hartl, Susanne Lezius, Peter Hübener, Kornelius Schulze, Roman Zenouzi, Marcial Sebode, Moritz Peiseler, Ulrike W. Denzer, Alexander Quaas, Christina Weiler-Normann, Ansgar W. Lohse, Olivier Chazouilleres, Christoph Schramm

**Affiliations:** 1 1st Department of Medicine, University Medical Center Hamburg-Eppendorf, Martinistr. 52, 20246 Hamburg, Germany; 2 Service d'Hépatologie, Centre de Référence des Maladies Inflammatoires des Voies Biliaires, Hôpital Saint-Antoine, Assistance Publique, Hôpitaux de Paris, Faculté de Médecine et Université Pierre et Marie Curie, site Saint-Antoine, Paris, France; 3 Service de Radiologie, Hôpital Saint-Antoine, Assistance Publique, Hôpitaux de Paris, Faculté de Médecine et Université Pierre et Marie Curie, site Saint-Antoine, Paris, France; 4 Department of Medical Biometry and Epidemiology, Martinistr. 52, 20246 Hamburg, Germany; 5 Department of Interdisciplinary Endoscopy, University Medical Center Hamburg-Eppendorf, Martinistr. 52, 20246 Hamburg, Germany; 6 Institute of Pathology, University Medical Center Hamburg-Eppendorf, Martinistr. 52, 20246 Hamburg, Germany; Yonsei University College of Medicine, REPUBLIC OF KOREA

## Abstract

**Background:**

Patients with primary sclerosing cholangitis (PSC) develop progressive liver fibrosis and end-stage liver disease. Non-invasive and widely available parameters are urgently needed to assess disease stage and the risk of clinical progression. Transient elastography (TE) has been reported to predict fibrosis stage and disease progression. However, these results have not been confirmed in an independent cohort and comparison of TE measurement to other non-invasive means is missing.

**Methods:**

In a retrospective study we collected data from consecutive PSC patients receiving TE measurements from 2006 to 2014 (n = 139). Data from 62 patients who also underwent a liver biopsy were used to assess the performance of TE and spleen length (SL) measurement for the staging of liver fibrosis. Follow-up data from this cohort (n = 130, Hamburg) and another independent cohort (n = 80, Paris) was used to compare TE and SL as predictors of clinical outcome applying Harrel’s C calculations.

**Results:**

TE measurement had a very good performance for the diagnosis and exclusion of higher fibrosis stages (≥F3: AUROC 0.95) and an excellent performance for the diagnosis and exclusion of cirrhosis (F4 vs. < F4: AUROC 0.98). Single-point TE measurement had very similar predictive power for patient outcome as previously published. In a combined cohort of PSC patients (n = 210), SL measurements had a similar performance as TE for the prediction of patient outcome (5 x cross-validated Harrel’s C 0.76 and 0.72 for SL and TE, respectively).

**Conclusions:**

Baseline TE measurement has an excellent performance to diagnose higher fibrosis stages in PSC. Baseline measurements of SL and TE have similar usefulness as predictive markers for disease progression in patients with PSC.

## Introduction

Primary sclerosing cholangitis (PSC) is characterized by the chronic inflammation of intra- and/or extrahepatic bile ducts, leading to biliary strictures and eventually biliary cirrhosis. Usually, PSC progresses to end-stage liver disease within 10–20 years [1]. To date, no medical treatment has proven to effectively alter the course of disease. PSC patients are at greatly increased risk to develop hepatobiliary carcinoma, mainly cholangiocarcinoma, which is associated with a dismal prognosis [2], however, for many patients morbidity and mortality is related to fibrosis progression to liver cirrhosis and its complications.

In 2003, the ultrasound-based method of transient elastography was introduced as a non-invasive means to measure liver fibrosis [3]. Since then liver stiffness measurement has developed into an important tool for the assessment of fibrosis in diseases such as viral hepatitis and non-alcoholic steatohepatitis [4–7]. Transient elastography is based on the assumption that the stiffness of the liver parenchyma can be measured as a surrogate parameter of fibrosis. TE has also been used to assess fibrosis in cholestatic liver diseases [8–10]. Liver stiffness may not only correlate with fibrosis stage, but also predict the patients´ clinical outcome; this has been shown for single measurements in chronic viral hepatitis [11,12] and non-alcoholic steatohepatitis [13] and also for serial measurements in patients with primary biliary cirrhosis [14]. Recently, it has been demonstrated in a single center study that baseline measurements and longitudinal changes could serve as prognostic factors for the clinical outcome in PSC [15]. However, these results have not been confirmed in an independent cohort. Moreover, we recently reported that the baseline measurement of the maximal diameter of the spleen by means of cross-sectional imaging such as ultrasound or magnetic resonance imaging (MRI) can be used to diagnose cirrhosis and stratify PSC patients according to their risks for clinical progression [16]. However, TE and SL measurement have not been compared in their ability to predict clinical progression in PSC.

We therefore aimed to analyse the usefulness of TE as a diagnostic tool for the stage of liver fibrosis and as a predictor of clinical outcome. Furthermore, we evaluated the test performance of SL- in comparison to TE measurement for the prediction of disease progression in two independent cohorts of patients with PSC.

## Materials and Methods

### Human subjects

Patients with PSC were diagnosed according to accepted criteria including typical findings in endoscopic retrograde cholangiopancreatography and/or magnetic resonance cholangiopancreatography [1]. TE was introduced to our institution in 2006. All patients with the diagnosis of PSC who were seen from 2006 until 2014 at the University Medical Center Hamburg-Eppendorf, Germany, were eligible for inclusion. PSC patients who already underwent liver transplantation before their first TE evaluation were excluded. Informed consent in writing was obtained from each patient and the retrospective analysis of patient data was approved by the Hamburg local ethical review board (OB-17/06, city of Hamburg, Germany) and conformed to the ethical guidelines of the 1975 Declaration of Helsinki.

Among 211 PSC patients screened, 130 had a valid TE measurement. 139 patients received a TE but 9 patients had to be excluded (3 patients had no TE result because of technical limitations due to obesity, 4 patients were excluded because the IQR/M was higher than 30%, 2 patients did not receive the minimum of 10 valid measurements). In 7 patients who had invalid tests at first, repeated measurements led to valid TE results. For the assessment of a possible prognostic value of TE in PSC, patients with at least one valid TE were included. Patients were followed-up until the endpoints liver related death, liver transplantation or hepatic decompensation (variceal bleeding, hepatic encephalopathy > grade I, ascites) were reached. Patients with a prior history of hepatic decompensation before their TE were excluded. The development of cholangiocarcinoma or gallbladder cancer were not included as an endpoint in the follow-up analysis, since these are not associated with fibrosis stage.

For the verification of SL as a predictor of the clinical outcome an independent cohort of PSC patients was evaluated. A total of 80 patients, in part included in a previously published cohort [15] had both, TE and MRI-based spleen length measurements. The spleen’s largest diameter (length, width or height) was utilized for calculations.

### TE and ultrasound

TE was performed with Fibroscan (Echosens, Paris, France) using the standard probe. We accepted a time interval of no longer than 6 months between liver biopsy and TE. If several TE results existed within this timeframe, the valid TE that was closest to the date of liver biopsy was chosen. At least 10 valid measurements were required and the TE results were expressed as the median and the IQR (plus IQR ratio). The IQR/M had to be less than 30%. Abdominal ultrasound was performed using the GE Logiq E9 and spleen length (SL) was determined by postero-lateral scanning, whereby the largest longitudinal diameter of the spleen was taken as SL. Measurements of TE in the Paris cohort has been published previously [15].

### Liver biopsy and histological evaluation

Eighty-four per cent of liver biopsies were performed under mini-laparoscopic guidance, as previously described [17]. In 66% of cases, both liver lobes were biopsied. In 3 cases a biopsy of the liver was obtained during surgery. The remaining liver biopsies were obtained under ultrasound guidance and were taken from the right liver lobe in standard technique using the Menghini needle. Liver fibrosis was staged according to Desmet and Scheuer [18] by an experienced liver pathologist (A.Q.). When staging results from both liver lobes gave divergent results, the higher stage was chosen for the comparison with TE values and for the analysis of disease outcome.

### Statistical analysis

Descriptive statistics are given as median and range. For the assessment of patient outcome, the endpoint free survival was calculated from baseline (the day when the patient underwent TE) until April 2014. The area under receiver operating characteristic (AUROC) was used for the analysis of the diagnostic performance of TE and SL. Sensitivity, specificity, positive and negative predictive values were determined for different cut-off points of TE and SL. Survival analyses were performed using the Cox proportional hazards regression. For graphical representation Kaplan-Meier curves are shown. For the comparison of the survival models according to the different classifications Harrell’s C was used [19]. To avoid overoptimism a cross validated Harrell’s C was calculated additionally. Due to the low number of events 5-fold cross validation was used. For all statistical tests a p-value below 0.05 was considered to be of statistical significance. The statistic calculations were performed using SPSS Statistics (IBM Corporation, Somers, New York), Stata analysis (StatCorp, Texas) software and Graphpad Prism (Graphpad Software Inc., La Jolla, California).

## Results

Patient characteristics and laboratory values at the time of TE and SL measurement are shown in Table 1. Patients had their TE measurement at the age of 38 years, a median of 2.3 years after the diagnosis of PSC. In total, the group of PSC patients with a valid TE comprises 130 patients of which 126 (97%) also received an ultrasound based SL measurement. Sixty-two of the patients with TE measurement underwent a liver biopsy of which 60 (97%) also received an ultrasound based SL measurement. The median time between liver biopsy and TE measurement was 1 month.

**Table 1:** Characteristics of PSC Patients.

Total number of PSC Patients reviewed	211
PSC patients with valid transient elastography	130
Age at Scan (years)	38 (16–75)
Male (%)	77 (59)
Time of TE after first diagnosis (months)	27 (0–319)
Time between TE and biopsy (months)	1 (0–6)
Liver Biopsy	62
Laboratory	
Alkaline Phosphatase (U/L)	146 (10–777)
Gamma Glutamyl Transferase (U/L)	130 (10–1398)
Total Bilirubin (mg/dL)	0.7 (0.3–10.3)
Albumin (g/dL)	43 (26–50)
Aspartate Aminotransferase (U/L)	38 (14–164)
Alanine Aminotransferase (U/L)	48 (8–407)
Creatinine (mg/dL)	0.8 (0.5–1.1)
International normalized ratio	0.99 (0.88–1.34)
Platelet Count (10^9^/L)	252 (46–549)

### Validation of liver stiffness measurement for the assessment of fibrosis stage in PSC

**Fig 1:**
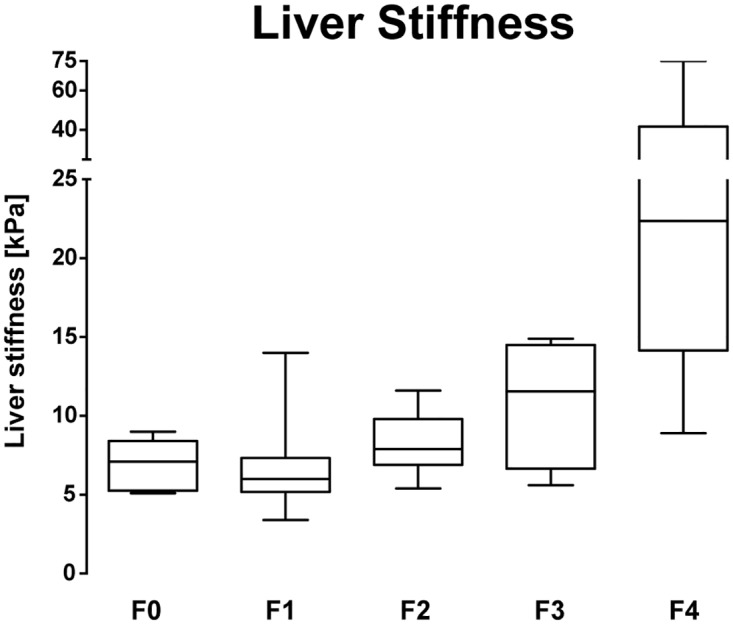
The Performance of TE Diagnosing Liver Fibrosis in PSC. Box plot of TE measurement depending on histological fibrosis stage F0-F4 (n = 62). The bottom and top of the boxes represent the 25^th^ and 75^th^ percentile and the horizontal lines the median. The whiskers are the minimum and maximum of the data.

We assessed patients who received a liver biopsy within six months of performing TE, and staged fibrosis according to the score of Desmet and Scheuer (n = 62). Most of the PSC patients had absent or minimal levels of fibrosis (F0-F1, 56.5%) whereas approximately one quarter showed liver cirrhosis (25.8%, Fig 1, Table 2). The median TE value for F1 fibrosis was 6 kPa and for F2 and F3 it was 7.9 kPa and 11.5 kPa, respectively. For liver cirrhosis (F4), the median TE value was 22.4 kPa (Fig 1, Table 2). For the diagnosis of liver cirrhosis, TE had an excellent overall performance (AUROC 0.978, Table 3, S1 Table). For the detection of lower fibrosis stages the results are summarized in table 3. Thus, TE proved to be useful for the detection and exclusion of higher fibrosis stages, whereas its test accuracy for lower stages of fibrosis was weaker. In the whole cohort (n = 130) TE results were correlated to standard laboratory values (S2 Table); however, we found the strongest correlation with spleen length (Spearman ρ = 0.52, p<0.0001).

**Table 2:** TE result and spleen length according to stage of fibrosis.

Patients with TE	Liver stiffness (kPa)	Length of spleen (mm)
F0, n = 5	7.1 (5.1–9)	95 (57–127)
F1, n = 30	6 (3.4–14)	106 (68–136)
F2, n = 7	7.9 (5.4–11.6)	116 (90–145)
F3, n = 4	11.5 (5.6–14.9)	126 (115–137)
F4, n = 16	22.4 (8.9–75)	155 (88–200)

**Table 3:** Cutoff Values and Performance of TE Measurement for the Diagnosis of Fibrosis Stage.

Stage	n	AUROC	95% CI	Cutoff (kPa)	SE	95% CI	SP	95% CI	PPV	95% CI	NPV	95% CI	ACC	95% CI
All patients	62													
**≥ F1**	57	**0.63**	0.44 to 0.82	**6.6**	**64.9**	52.5 to 77.3	**60**	17.1 to 100	**94.9**	87.9 to 100	**13**	0 to 26.8	**64.5**	52.6 to 76.4
**≥ F2**	27	**0.91**	0.82 to 0.99	**8.8**	**81.5**	66.8 to 96.1	**88.6**	78 to 99.1	**84.6**	70.7 to 98.5	**86.1**	74.8 to 97.4	**85.5**	76.7 to 94.3
**≥ F3**	20	**0.95**	0.89 to 1.0	**9.6**	**90**	76.9 to 100	**90.5**	81.6 to 99.4	**81.8**	65.7 to 97.9	**95**	88.2 to 100	**90**	83 to 97.7
**F4**	16	**0.978**	0.93–1.0	**14.4**	**68.8**	46 to 91.5	**97.8**	93.6 to 100	**91.7**	76 to 100	**90**	81.7 to 98.3	**90.3**	83 to 97.7

AUROC Area under the receiver operator characteristic curve, SE sensitivity, SP specificity, PPV positive predictive value, NPV negative predictive value, ACC accuracy, CI confidence interval.

**Fig 2:**
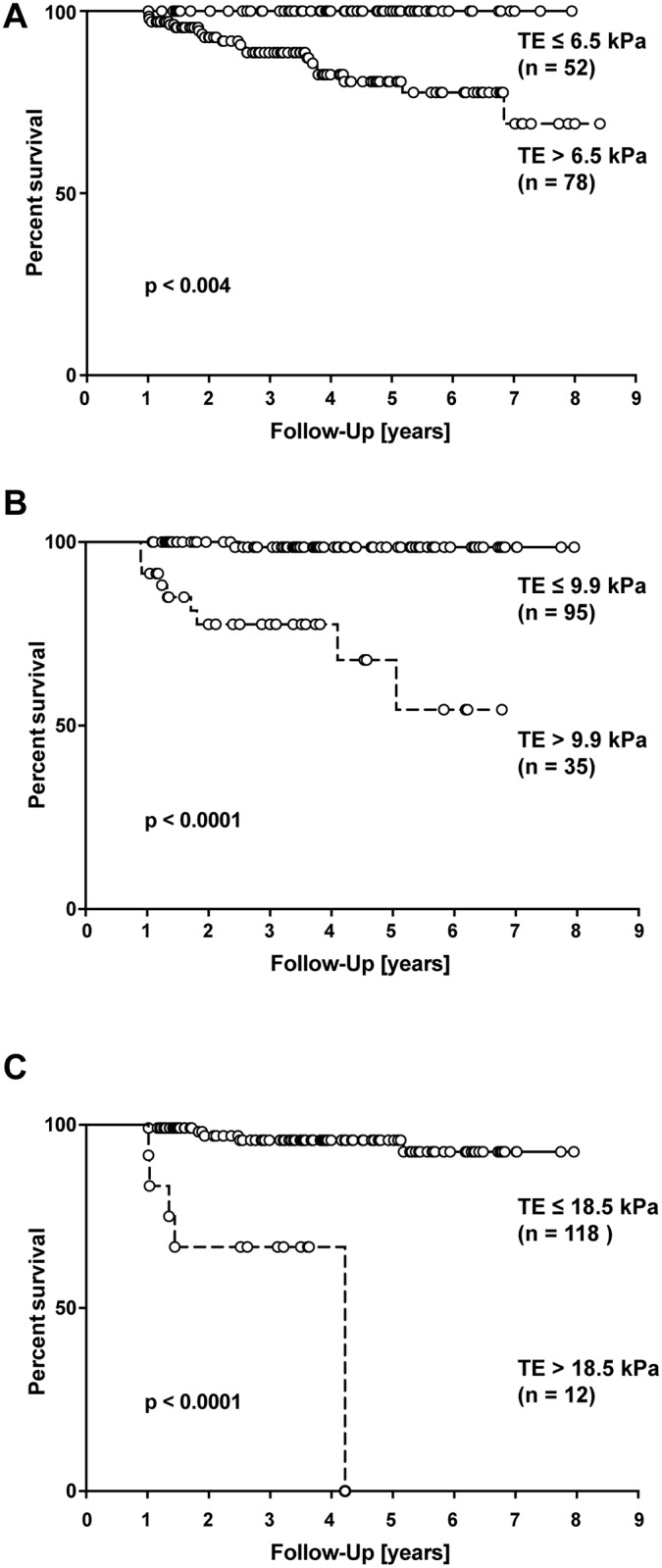
Survival Rates of PSC Patients for Different TE Cut-off Values. Survival rates according to the different cut-off values (A-C) of TE measurement as previously suggested [15]. (log-rank test, p<0.0001).

Next, we sought to evaluate TE measurement as a predictor of the clinical outcome in our cohort of PSC patients. One hundred thirty PSC patients met the inclusion criteria and were followed-up for a median of 43 months (range from 12 to 97 months). For the previously published liver stiffness cut-off values of 6.5 kPa, 9.9 kPa and 18.5 kPa [15] survival rates are depicted in Fig 2. TE results were strongly associated with the risk of liver transplantation, death or hepatic complications for any of the cut-offs investigated, which confirmed the previously published data [15]. The optimal cut-off for this cohort was 12.4 kPa (Youden index).

### Comparison of TE and SL measurement as predictors of clinical outcome

**Fig 3:**
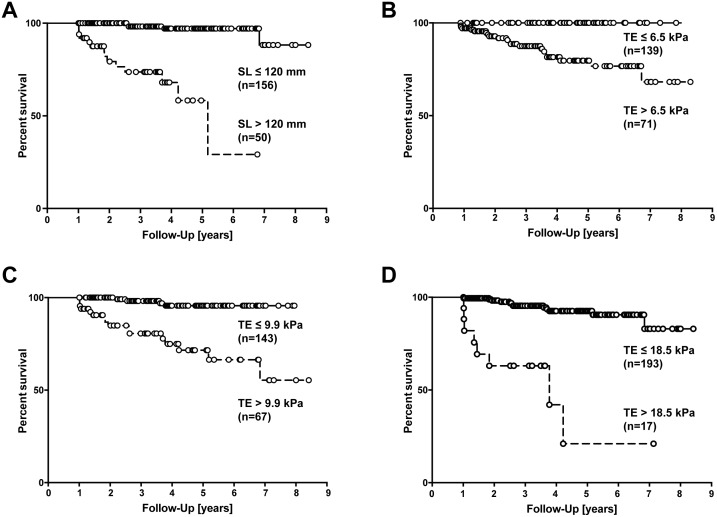
Survival rates according to SL and TE measurement. Survival rates according to SL measurement with 120 mm cut-off and according to TE measurement with different cut-off values (B-D) for the combined cohort of PSC patients (Hamburg + Paris, see text). (log-rank test, p<0.001 each).

We recently reported that SL measurement can be used as a predictor of clinical prognosis in patients with PSC [16]. To compare the predictive power of TE with SL measurement we now combined the two previously published cohorts (Hamburg + Paris, n = 210, Table 4). For this combined group of patients, a cut-off of 120 mm spleen length was strongly associated with the risk of liver transplantation, death or hepatic complications (Fig 3A). Using cross-validated Harrel’s C calculations we found that SL measurement with the proposed cut-off of 120 mm was not inferior to any TE measurement cut-off when used as a predictor of clinical outcome (Table 5, Fig 3A–3C). When we compared outcomes in a multivariate Cox analysis, both TE and spleen length were statistically significant predictors of outcome. These data suggest that single point measurements of TE and SL can be used equally well to stratify PSC patients according to the risk of clinical progression.

**Table 4:** Characteristics of PSC Patient Cohorts.

	*Hamburg*	*Paris*	*Combined*
Number of PSC patients	130	80	210
Age, years (range)	38 (16–75)	40 (14–72)	39 (14–75)
Male (%)	59.20	67.50	62.40
Follow Up, months (range)	43 (12–97)	46 (12–102)	44 (12–102)
Endpoints, n (%)	10 (7.7)	10 (12.5)	20 (9.5)
TE, kPa (range)	7.7 (2.5–75)	8.2 (3.9–73.5)	7.8 (2.5–75)
Spleen length, mm (range)	111 (57–200)	103 (24–205)	109 (24–205)

TE transient elastography.

**Table 5:** Comparison of SL and TE in predicting clinical outcome.

	HR (95%CI)	Harrel's C	5 x cv Harrel's C
SL > 120 mm	14.2 (5.0–40.6)	0.798	0.757
TE > 8.1 kPa	12.7 (2.9–54.8)	0.748	0.690
TE > 9.9 kPa	9.5 (3.2–28.5)	0.765	0.716
TE > 18.5 kPa	9.7 (3.9–23.8)	0.719	0.672

SL spleen length, TE transient elastography, CI confidence interval, HR hazard ratio, 5x cv 5 times cross-validated.

## Discussion

In the majority of cases PSC is a progressive disease that ultimately leads to complications related to portal hypertension and cirrhosis. Until today, no medical treatment has demonstrated to improve the dismal course of PSC. Novel drugs targeting fibrogenesis and inflammation are increasingly being tested in PSC. One of the major current needs in PSC is to define surrogate endpoints for clinical trials [20]. Additionally, a simple, widely available and non-invasive point of care tool for risk-assessment would aid patient counselling and care in clinical practice. TE has recently been suggested as a non-invasive test for the staging of fibrosis in PSC and, more importantly, as a marker of clinical progression [15]. These data, however, have so far not been confirmed in an independent cohort of PSC patients.

The data presented here confirm the usefulness of TE measurement as a diagnostic tool for the presence or exclusion of liver cirrhosis in PSC. For the Hamburg cohort we found an AUROC very similar to the data recently published [15]. We also found that TE had an overall very good test accuracy for fibrosis stages F2 and F3, whereas it does not allow the differentiation of lower stages of liver fibrosis. Importantly, our data strongly support the value of TE for the assessment of the clinical progression of PSC. Using the suggested cut-offs resulted in a risk stratification of patients very similar to the data published previously [15]. Of note, in accordance with the previously published data [15] we confirm a high accuracy of TE diagnosing higher stages of fibrosis (≥ F3) for liver stiffness reaching > 9.5 kPa. This is an important observation taking into consideration that previous work strongly suggested that fibrosis progression accelerates significantly once this stage has been reached [15].

Spleen length correlates well with portal hypertension and grade of liver fibrosis. SL measurements can easily be performed during ultrasound exams or other cross sectional imaging techniques with a very low technical failure rate. We recently reported that SL measurement can be used to diagnose liver cirrhosis and for risk stratification according to the clinical outcome in patients with PSC [16]. For the diagnosis of liver cirrhosis TE measurement demonstrated a better performance than measurement of SL. In particular, the specificity, negative predictive value (NPV) and positive predictive value (PPV) for TE appeared better than for SL measurements [15,16]. In our centre we try to minimize the bias introduced by the focal nature of PSC by performing laparoscopically guided biopsies. Although there are differences in fibrosis stage in around 10% of biopsies from left and right liver lobes, this did not significantly influence the results reported here (data not shown).

The value of SL as a prognostic marker in comparison to TE in patients with PSC has not been addressed so far. Our data show that single point TE and SL measurement show a similar performance in predicting disease progression in PSC. Importantly, for clinical progression of PSC and PBC it has been shown that changes of the TE value over time stratified patients effectively according to their risk of progression [14,15]. In the study reported here, only single TE and SL measurements were assessed. Nevertheless, we clearly see patients who progress quite rapidly in liver stiffness and spleen length whereas others remain stable over years. It will be interesting to analyse if the evaluation of serial SL measurements will further increase the capacity to predict disease progression.

As an endpoint we had defined all liver related deaths excluding deaths related to cholangiocarcinoma (CCA) and gallbladder cancer (GBC), since it has been shown that the incidence of these malignancies was not related to disease stage in PSC [1,21,22]. In the combined cohort of PSC patients there were five patients with endpoints related to hepatobiliary malignancy, which were not included in the analysis: 4 patients developed CCA and had TEs ≤ 7.9 kPa and SLs ≤ 116 mm and one patient developed gallbladder cancer and died. These data confirm that TE and SL measurements fail to predict a poor prognosis due to biliary malignancy.

Besides obvious limitations of a retrospective study design, confounding factors that may influence liver stiffness such as cholestasis [23] and inflammation [24] must be taken into account and may specifically impact on TE values in patients with PSC. The data reported here demonstrated a weak correlation between TE values and serum bilirubin levels as a marker of bile duct obstruction (S2 Table). However, serum bilirubin levels also increase in liver cirrhosis, making it impossible to differentiate between fibrosis stage and bile duct obstruction. We therefore investigated, whether high bilirubin levels in patients without liver cirrhosis influenced TE measurements: in patients with stage 0-III disease, bilirubin levels did not correlate with TE values (data not shown). Since it has been described that intrahepatic cholestasis may increase liver stiffness [23] it seems prudent to exclude bile duct dilatation of the right liver lobe prior to elastography [25].

It is now standard at our center to measure liver elastography in all patients with PSC on a regular basis. One limitation of our retrospective study is, that in the early days of transient elastography some patients, mainly with advanced disease were missed for reasons such as direct referral to our transplant unit. It is also important to note, that 70% of the endpoints occurred among individuals already known to have cirrhosis and a larger cohort of patients is required to accurately describe the performance of TE/spleen length among individuals who do not have cirrhosis at baseline.

PSC can be accompanied with a varying degree of liver inflammation. For our cohort, we found only weak correlations of TE and SL results both with the levels of serum aminotransferases and with the modified hepatitis activity index (data not shown). Since the presence of liver cirrhosis influences aminotransferase levels as well as liver inflammation, we separately analysed the correlation of TE and SL values with hepatic inflammation in non-cirrhotic patients. In these patients, there was no significant correlation between TE values and liver inflammation.

For the interpretation of TE results it should be kept in mind that by the limitation of the technique only a small volume of the right liver lobe can be measured and that the disease is focal with high variability of affection between liver segments. Spleen size may circumvene this problem and reflect sum changes of inflammation, fibrosis and portal hypertension, and enlargement of the spleen can be seen in single PSC patients already at an early disease stage. In addition, we observed a failure rate for TE of 6%, whereas the spleen length could be obtained by ultrasound or MRI in all patients but one who had asplenia. However, TE failure rate might significantly decrease with the use of larger probes that were not used in this study.

Other biomarkers of liver fibrosis, such as the enhanced liver fibrosis (ELF) score [26] or magnetic resonance elastography [27] are under evaluation as markers of disease stage and progression. The ELF score was also found to correlate with ultrasound elastography, however, to date, a direct comparison of different techniques has not been performed.

In conclusion, we here confirm the value of TE for the diagnosis of cirrhosis and the prediction of PSC disease course. SL and TE can both be used to accurately diagnose liver cirrhosis, but TE measurement may be superior to SL for cirrhosis exclusion. Single point SL was shown to be equal to single point TE measurement for the prediction of disease progression in PSC.

## Supporting Information

S1 Table Performance of TE Measurement for the Diagnosis of Cirrhosis (F4) for Different Cut-off Values. (DOCX)

S2 Table Correlation of Transient Elastography with Standard Laboratory Markers and Spleen Length. (DOCX)
