# Supplementary material for: Validation of Transient Elastography and Comparison with Spleen Length Measurement for Staging of Fibrosis and Clinical Prognosis in Primary Sclerosing Cholangitis
Source: PLoS One. 2016 Oct 10;11(10):e0164224. doi: 10.1371/journal.pone.0164224 (PMC5056739; doi:10.1371/journal.pone.0164224)
Supplement: S1 Table — (DOCX) [file pone.0164224.s001.docx]

| **Supplemental Table 1. Performance of TE measurement for the diagnosis of cirrhosis (F4) for different cut-off values** | | | | | | |
| --- | --- | --- | --- | --- | --- | --- |
| **AUROC** | **Cut-off** | **Sensitivity** | **Specificity** | **PPV** | **NPV** | **Accuracy** |
| (95% CI) |  | (95% CI) | (95% CI) | (95% CI) | (95% CI) | (95% CI) |
|  |  |  |  |  |  |  |
|  | (kPa) |  |  |  |  |  |
| 0,978 (0.934-1.008) | 8.9 | 100 | 80 (69-92) | 64 (45-83) | 100 | 86 (76-94) |
|  | 12.3 | 94 (82-100) | 96 (90-100) | 88 (73-100) | 98 (94-100) | 95 (89-100) |
|  | 14.1 | 81 (62-100) | 98 (94-100) | 93 (79-100) | 94 (87-100) | 94 (87-100) |
|  |  |  |  |  |  |  |
|  |  |  |  |  |  |  |
|  |  |  |  |  |  |  |
|  |  |  |  |  |  |  |
