# Supplementary material for: Validation of Transient Elastography and Comparison with Spleen Length Measurement for Staging of Fibrosis and Clinical Prognosis in Primary Sclerosing Cholangitis
Source: PLoS One. 2016 Oct 10;11(10):e0164224. doi: 10.1371/journal.pone.0164224 (PMC5056739; doi:10.1371/journal.pone.0164224)
Supplement: S2 Table — (DOCX) [file pone.0164224.s002.docx]

| **Supplemental Table 2. Correlation of Transient Elastography with Standard Laboratory Markers and Spleen Length** | | | | | | | | | | | |
| --- | --- | --- | --- | --- | --- | --- | --- | --- | --- | --- | --- |
|  | **Albumin** | **AP** | **GGT** | **ALT** | **AST** | **INR** | **Bilirubin** | **Creatinine** | **Platelet count** | **Length of spleen** | **AST/PLT** |
|  |  |  |  |  |  |  |  |  |  |  |  |
| Number of test pairs | 104 | 114 | 119 | 121 | 116 | 104 | 119 | 103 | 119 | 112 | 110 |
| Spearman r | -0.180 | 0.448 | 0.319 | 0.349 | 0.444 | 0.248 | 0.299 | -0.124 | -0.220 | 0.504 | 0.433 |
| 95% CI | -0.36 - 0.0253 | 0.262 - 0.572 | 0.1572 - 0.488 | 0.207 - 0.524 | 0.250 - 0.562 | 0.068 - 0.438 | 0.147 - 0.48 | -0.252 - 0.145 | -0.385 - -0.031 | 0.289 - 0.594 | 0.223 - 0.549 |
| P value | 0.08 | < 0,0001 | 0.0002 | < 0,0001 | < 0,0001 | 0.01 | 0.0003 | 0.57 | 0.02 | < 0,0001 | < 0,0001 |
|  |  |  |  |  |  |  |  |  |  |  |  |
|  |  |  |  |  |  |  |  |  |  |  |  |
